# Web-Based Mindfulness Intervention in Heart Disease: A Randomized Controlled Trial

**DOI:** 10.1371/journal.pone.0143843

**Published:** 2015-12-07

**Authors:** John O. Younge, Machteld F. Wery, Rinske A. Gotink, Elisabeth M. W. J. Utens, Michelle Michels, Dimitris Rizopoulos, Elisabeth F. C. van Rossum, M. G. Myriam Hunink, Jolien W. Roos-Hesselink

**Affiliations:** 1 Department of Cardiology, Erasmus MC, University Medical Center Rotterdam, Rotterdam, The Netherlands; 2 Department of Epidemiology, Erasmus MC, University Medical Center Rotterdam, Rotterdam, The Netherlands; 3 Department of Psychiatry (Section Medical Psychology and Psychotherapy), Erasmus MC Rotterdam, University Medical Center, Rotterdam, The Netherlands; 4 Department of Adolescent Psychiatry/Psychology, Erasmus MC Rotterdam, University Medical Center, Rotterdam, The Netherlands; 5 Department of Radiology, Erasmus MC, University Medical Center Rotterdam, Rotterdam, The Netherlands; 6 Department of Biostatistics, Erasmus MC, University Medical Center Rotterdam, Rotterdam, The Netherlands; 7 Department of Internal Medicine, Erasmus MC, University Medical Center Rotterdam, Rotterdam, The Netherlands; 8 Department of Health Policy and Management, Harvard T.H. Chan School of Public Health, Boston, MA, United States of America; University of Glasgow, UNITED KINGDOM

## Abstract

**Background:**

Evidence is accumulating that mindfulness training has favorable effects on psychological outcomes, but studies on physiological outcomes are limited. Patients with heart disease have a high incidence of physiological and psychological problems and may benefit from mindfulness training. Our aim was to determine the beneficial physiological and psychological effects of online mindfulness training in patients with heart disease.

**Methods:**

The study was a pragmatic randomized controlled single-blind trial. Between June 2012 and April 2014 we randomized 324 patients (mean age 43.2 years, 53.7% male) with heart disease in a 2:1 ratio (n = 215 versus n = 109) to a 12-week online mindfulness training in addition to usual care (UC) compared to UC alone. The primary outcome was exercise capacity measured with the 6 minute walk test (6MWT). Secondary outcomes were other physiological parameters (heart rate, blood pressure, respiratory rate, and NT-proBNP), subjective health status (SF-36), perceived stress (PSS), psychological well-being (HADS), social support (PSSS12) and a composite endpoint (all-cause mortality, heart failure, symptomatic arrhythmia, cardiac surgery, and percutaneous cardiac intervention). Linear mixed models were used to evaluate differences between groups on the repeated outcome measures.

**Results:**

Compared to UC, mindfulness showed a borderline significant improved 6MWT (effect size, meters: 13.2, 95%CI: -0.02; 26.4, p = 0.050). There was also a significant lower heart rate in favor of the mindfulness group (effect size, beats per minute: -2.8, 95%CI: -5.4;-0.2, p = 0.033). No significant differences were seen on other outcomes.

**Conclusions:**

Mindfulness training showed positive effects on the physiological parameters exercise capacity and heart rate and it might therefore be a useful adjunct to current clinical therapy in patients with heart disease.

**Trial Registration:**

Dutch Trial Register 3453

## Introduction

In recent decades, cardiovascular disease (CVD) has become the foremost cause of health burden worldwide.[1] Especially the group of adults with congenital heart disease has increased over the last decades. While cardiovascular disease cause significant stress,[2] chronic stressors such as anxiety and depression are themselves independent risk factors for cardiovascular morbidity and mortality.[3, 4] Chronic stress can negatively affect not only quality of life, but also physiological parameters such as respiration rate, heart rate, blood pressure, inflammatory markers and brain activity.[5]

As heart rate is associated with long-term survival, patients are recommended to try reducing heart rate in the management and prevention of CVD.[6] Often medication, such as betablockers, is prescribed for this goal. Stress reduction in itself may also have a beneficial effect on heart rate and physical fitness. While the best approach to stress management is unclear, increased attention is now being paid to lifestyle interventions such as mindfulness therapy.[7, 8] Mindfulness is described as the capacity to live with open and non-judgmental awareness towards all experiences within the present moment.[9, 10] Several core features, such as meditation, yoga, and cognitive assignments, can increase the ability to accept negative experience or emotions.[11] Mindfulness therapy has been found to positively affect psychological outcomes in patients with chronic pain, obesity, hypertension, depression, anxiety and cardiovascular disease.[12–16]

We hypothesized that, besides these psychological effects, mindfulness therapy may influence heart rate, breathing patterns and blood pressure through a favorable effect on the autonomic nervous system and therefore may positively affect exercise capacity and thus long-term outcome[17]. In a randomized controlled trial (RCT), we therefore investigated the effectiveness of online mindfulness training on exercise capacity in patients with heart disease.

## Methods

### Study design

The current study is a single blinded, pragmatic RCT performed at the outpatient cardiology clinic of the Erasmus MC, Rotterdam, The Netherlands. Ethical approval was obtained from the Medical Ethics Committee (METC) of the Erasmus Medical Center and the study complied with the Declaration of Helsinki (S1 and S2 Texts). The study was registered at the Dutch trial register, 3453, http://www.trialregister.nl. Patients received written information about the study at home, 2–4 weeks prior to their scheduled visit to the cardiologist at the outpatient clinic. Full disclosure was given about the nature of the intervention. The current study reports the results of 3 month follow-up, which ended in July 2014, whereas the 12-month follow-up is still ongoing.

### Participants

Adult patients, between 18 and 65 years of age, with diagnosed heart disease (ischemic, valvular, congenital heart disease, or cardiomyopathy) were eligible for inclusion between June 2012 and April 2014. Patients were excluded based on the following criteria: (1) planned operation or percutaneous intervention within the upcoming year; (2) inability or unwillingness to give informed consent; (3) inability to understand Dutch, inability to read or write Dutch; (4) no internet access, email, or cell phone; (5) patients who did not fill out the baseline questionnaires or did not show up for the scheduled baseline tests. All participants provided written informed consent.

### Intervention

The active intervention was mindfulness training which consisted of a 12-week structured standardized online program. The training was offered in addition to usual care (UC) as provided by the treating cardiologist. All patients received a book about mindfulness by a renowned author to support the 12-week training[18]. The training was designed to be self-directed and to be easily accessible and engaging to a wide audience by keeping practice sessions and lessons short. The program teaches different meditations, self-reflection, and yoga. Furthermore, it includes practical assignments and suggestions for mindfulness in day-to-day life. The use of breath as a reminder for present moment awareness is emphasized in all meditations. The program was divided into four components (S1 Table). During the course participants also received biweekly reminders by e-mail and standardized text messages. Adherence to the intervention was monitored by whether the questions of the online program were completed. For privacy reasons, the content of the answers remained undisclosed. Both the program and the book were provided free of charge to participating patients.

### Control

The control group received UC by their treating cardiologist. Treatment and frequency differs between patients, but general components are regular outpatient visits, lifestyle advice regarding nutrition, smoking, exercise, stress reduction, medication and other procedures if indicated. We chose for a pragmatic study design without a placebo online training in order to measure effectiveness rather than efficacy. This choice is justified by the likelihood of a partial placebo effect that is part-and-parcel of the training as it would be implemented in future practice.

### Randomization

After a patient’s eligibility was established by one of the study investigators, written informed consent was obtained and baseline measurements were performed. Subsequently, patients were randomized according to a 2:1 ratio via dedicated computer software (ALEA®) with a block size of 12 to receive the online Mindfulness training or UC.[19] The investigator entered the patients into the computer software, but did not receive the result of the allocation. The result of the randomization procedure was sent to an independent employee (medical secretary) of the outpatient clinic, who was not involved in establishing eligibility, outcome assessment, or data analyses. Subsequently, this employee contacted the participant with the result and provided instructions on how to access the web-based training.

### Blinding

Due to the nature of the intervention, blinding of patients was not feasible. The intervention started as soon as patients logged on to the mindfulness training website. The outcome assessors (investigators) were unaware of patients’ treatment allocation. Therefore, the design of this study can be considered as a single-blinded randomized controlled trial in which the investigators remained blinded throughout the duration of the study. Additionally, patients were instructed not to say anything about their treatment allocation, neither to study investigators nor to their cardiologist.

### Outcome measures

Outcomes were measured in all patients pre- (T0) and post-intervention (12 weeks, T1).

We were interested in evaluating the physical effects of mindfulness and thus chose as primary outcome measure the 6MWT, which is an overall measure of exercise tolerance, has reproducible results, and has shown to be an independent predictor of long term outcome.[20–22] The 6MWT was performed in a 20-meter-long corridor at the outpatient clinic.[23] The corridor had well-indicated ‘start’ and ‘finish’ marks with colored pawns.

### Secondary outcome measures

#### Physical parameters

Weight, blood pressure, respiratory rate and heart rate. BMI was calculated by weight in kilograms divided by height in meters squared. Blood pressure was measured using an automated non-invasive monitor (Mindray Datascope Duo) after the participant had rested for 5 minutes in the sitting position. This monitor also reports the heart rate. Respiratory rate was measured in rest within a set amount of 30 seconds.

#### Blood sampling laboratory tests

N-terminal pro-brain natriuretic peptide (NT-proBNP, Elecsys system, Roche Diagnostics, Basel, Switzerland: normal values ≤ 14pmol/L) and creatinin were measured from peripheral venous blood samples.

#### Subjective health status

The Short-Form Health survey 36 (SF-36) was used to evaluate subjective health status. For each of the 8 subdomains a transformed score is generated, ranging from 0 to 100,[24] with a higher score indicating better health.[25] The subdomains were used to construct the mental component summary (MCS) measure, which consists of the subdomains vitality, social functioning, role-emotional functioning and mental health, and the physical component summary (PCS) measure, which consists of the subdomains physical functioning, role physical functioning, bodily pain and general health.[26]

A Visual Analogue Scale (VAS) was used to assess subjective perceived QoL (“Indicate on the line above where you would situate yourself in terms of your overall quality of life”, ranging from 0 to 100, with a higher score indicating better QoL.[27]

#### Psychological well-being

To assess symptoms of anxiety and depression, the Hospital Anxiety and Depression scale was used. The questionnaire contains 14 items on depression and anxiety with a higher score on the 3 point Likert scale indicating a greater level of emotional distress.[28]

#### Stress

The Dutch version of the Perceived Stress Scale (PSS) was used to evaluate perceived stress. The scale consists of fourteen 5-point Likert scales, with a higher score indicating a higher level of stress (0 = never, 4 = very often). A total perceived stress score is made by summing all individual items.[29]

#### Social support

To evaluate perceived social support, the Dutch version of the Perceived Social Support Scale 12 Blumenthal (PSSS12) was used. The PSSS12 has 12 items with a 7-point Likert scale addressing the degree of perceived social support with a higher score indicating a greater feeling of support (1 = very strongly disagree, 7 = very strongly agree).[30] For the purpose of this study we used the total score.

#### Adverse events

Adverse events were defined as all-cause mortality, heart failure, symptomatic arrhythmia, cardiac surgery, and percutaneous cardiac intervention. Arrhythmias were defined as symptomatic if antiarrhythmic medication was prescribed, cardioversion or ablation had been applied, or a pacemaker or intracardiac defibrillator (ICD) was implanted. Heart failure was defined as an event when either medication or hospitalization was necessary.

### Other study parameters

In order to document baseline risk levels, traditional cardiovascular risk factors and demographics were determined: age, sex, length, weight, smoking, type of heart disease, and employment status. Additionally participation in other mindfulness-based exercises and the use of other complementary care was monitored with a questionnaire (type, frequency, and intensity).

### Quality control and audit

The digitalization of the paper case record forms (CRFs) in the database was independently performed by 2 persons (JY and MW). After digitalization, an error rate of <0.5% was observed between JY and MW. An independent audit was performed and the study was found to comply with Good Clinical Practice and Scientific Integrity standards.

### Sample size justification

To demonstrate an improvement of 5% in the intervention group vs 1% in the control group on the 6MWT, this study required 99 patients in the control group and 198 in the active intervention group (SD10%, alpha = 0.05, power = 0.90, ratio experimental to controls = 2). Even if only 50% of patients in the experimental group adhered to the training, this would give us a power of 0.80 in the as-treated analysis. To account for non-adherence and loss to follow-up our aim was to randomize at least 300 patients. This number of patients is sufficient to demonstrate a smaller difference (5% in the intervention group vs 2% in the control group) in a repeated measurements analysis with a power of 75% (2 follow-up measurements, correlation between follow up measurements = 0.70, correlation between baseline & follow-up = 0.50).

### Statistical analysis

Descriptive analyses were performed to describe the baseline characteristics of demographic and clinical variables stratified by treatment group. Changes in outcomes at 12 weeks compared with baseline (in**tra**group effect) and differences between treatment groups (difference in delta’s, in**ter**group effect) on physiological and psychological outcomes were calculated. To simultaneously account for the correlation between the multiple measurements of each patient and dropout, a repeated measurements analysis was performed using a multivariate linear regression mixed model to determine intergroup effects. In the mean structure of the mixed model we included the time effect, the intervention effect and their interaction, while a fully unstructured variance-covariance matrix was assumed for the error terms. Due to randomization only p-values for the interaction effect are reported.

An intention-to-treat (ITT) analysis was performed to address whether offering a mindfulness training was effective compared to UC. An as-treated (AT) analysis was performed to address whether the mindfulness training was beneficial if actually performed. In the AT analysis, patients were considered adherent if they completed 50% or more of the exercises. Patients allocated to the UC group who sought mindfulness training on their own were excluded from the AT analysis.

For both ITT and AT, Cohen’s D was calculated to enable comparison of effect sizes. This calculation was performed based on the results of the mixed model.

A p-value less than 0.05 was considered to be indicative of statistical significance. All data were analyzed with IBM SPSS Statistics version 21.0 (IBM Corp., Somers, NY).

## Results

### Patient recruitment and characteristics

**Fig 1:**
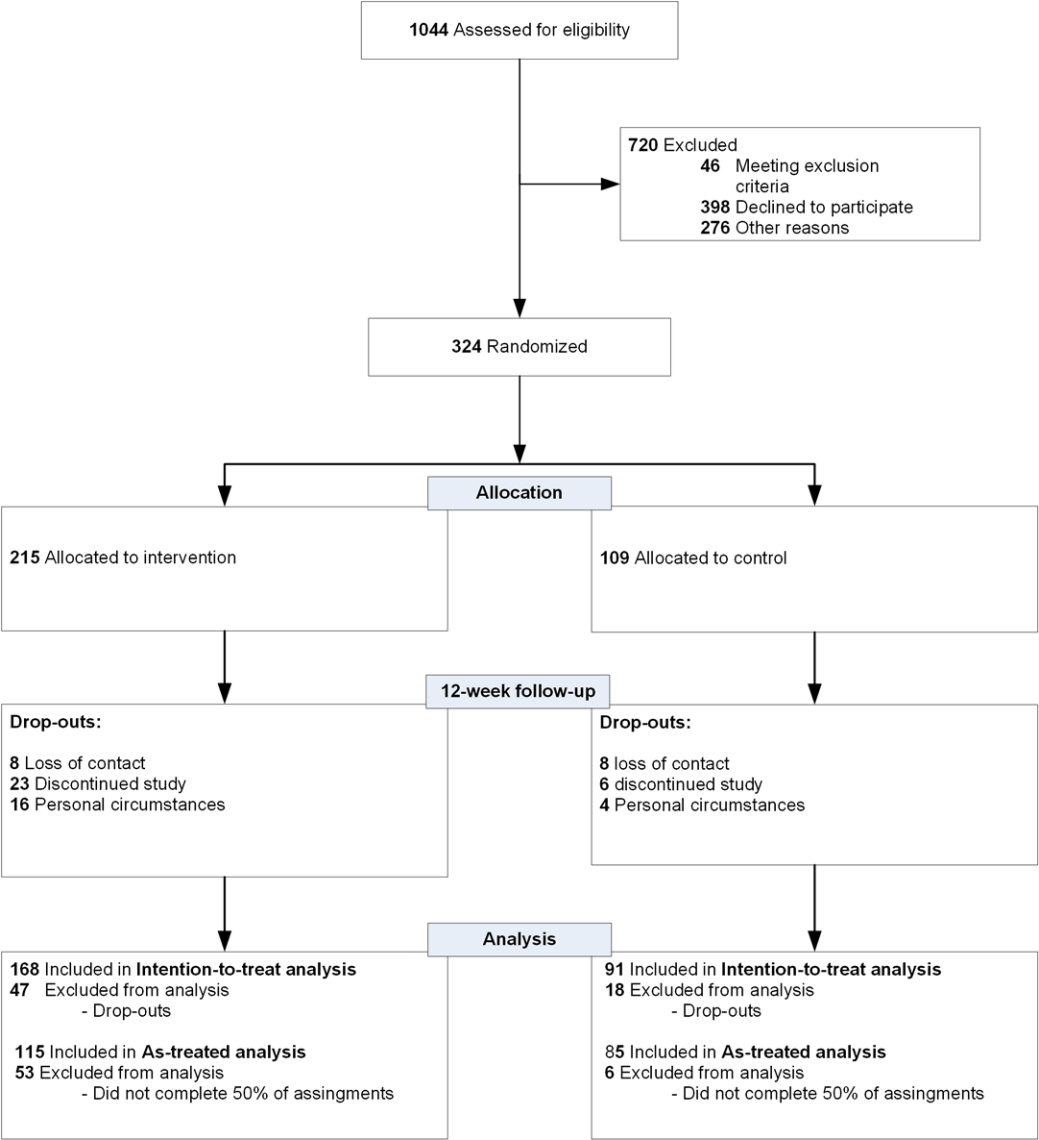
Flowchart of mindfulness training and control group.

A flowchart of the patients’ recruitment is shown in Fig 1. Patients’ baseline characteristics (Table 1) demonstrated no significant differences between the intervention and control group which confirmed a successful randomization, also on important characteristics such as: age (p = 0.98) and, gender (p = 0.28). In total, 5 patients did not complete any assignment of the mindfulness training whereas 115 patients completed at least 50% of the assignments (as-treated analysis) with a mean (SD) 53% (34).

**Table 1:** Baseline characteristics of study participants.

		Mindfulness Group N = 215	Control Group N = 109
Demographics			
Age (years), mean (SD)		43.2 (14.1)	43.2 (13.7)
Female (%)		44.2	50.5
Physiological parameters			
Heart rate (beats/min), mean (SD)		68 (12)	69 (11)
Systolic blood pressure (mm Hg), mean (SD)		128 (16)	125 (15)
Diastolic blood pressure (mm Hg), mean (SD)		78 (11)	80 (10)
Resting respiratory rate (breaths/min), median (IQR)		15 (2)	15 (3)
Body mass index (kg/m^2^), mean (SD)		25.9 (4.6)	25.7 (4.7)
Obesity* (%)		16.7	15.6
Psychological parameters			
PCS, mean (SD)		46.6 (9.6)	45.3 (10.3)
MCS, mean (SD)		50.2 (10.6)	50.8 (9.6)
HADS Anxiety, mean (SD)		8.2 (3.6)	9.0 (3.4)
HADS Depression, mean (SD)		3.8 (2.9)	3.8 (2.9)
VAS, mean (SD)		75.0 (13.2)	72.7 (13.2)
PSS, mean (SD)		22.4 (7.8)	22.0 (7.5)
PSSS12, mean (SD)		69.5 (11.6)	71.5 (12.3)
Exercise tolerance			
6 minute walk test distance (meters), mean (SD)		537.5 (77.0)	539.3 (67.3)
Laboratory works			
NT-proBNP, median (IQR), pmol/L		16.7 (28.5)	18.3 (33.9)
Creatinine, median (IQR), μmol/L		79.0 (21.0)	77.0 (21.0)
Cardiac history, type of heart disease, (%)			
Congenital heart disease		41.9	42.2
Cardiomyopathy		39.5	29.4
Valvular heart disease		18.6	28.4
Other comorbidity, (%)			
Diabetes Mellitus**		3.2	3.7
Number of interventions in life***, mean (SD)		1.4 (1.4)	1.4 (1.2)
Time since first intervention (years), mean (SD)		19.1 (14.0)	15.9 (11.7)
ICD, (%)		5.9	*4*.*3*
PM, (%)		9.3	5.2
Current medication (%)		70.2	72.5
Beta-blocker		43.2	36.7
Statin		18.6	13.8
Aspirin		16.3	14.7
Ace-inhibitor		23.3	22.0
Angiotensin II antagonist		8.8	11.9
Calcium channel blocker		9.8	6.4
Nitroglycerin		2.3	0.0
Cardiac glycoside		2.3	2.8
Diuretic		16.8	19.3
Anticoagulant		24.6	33.9
Antidepressant		5.1	2.8
Tranquilizer		1.9	1.8
Other		43.3	57.8
Intoxication, (%)			
Current smoking		14.4	18.3
Current alcohol use		62.1	55.0
Current drugs use		3.3	2.8
Work status			
Employed, (%)		68.7	67.9
Prior use of complementary therapies****, (%)		14.4	12.8

* Obesity was defined when the BMI was ≥30 kg/m2.

** Diabetes was defined when a patient reported use of anti-diabetes medication.

*** Include both surgical and percutaneous interventions.

**** Contains yoga, meditation, mindfulness, tai chi, Qigong and acupuncture.

SD, standard deviation; PCS, physical component summary measure; MCS, mental component summary measure; VAS, visual analogue scale; HADS, hospital anxiety and depression scale; PSS, perceived stress score; PSSS12, perceived social support; NT-proBNP, N-terminal pro-Brain Natriuretic Peptide; IQR, interquartile range; ICD, implantable cardioverter-defibrillator; PM, pacemaker.

### Safety/side effects

No major side effects were reported during the follow-up period. In 7 patients (5 mindfulness (2.3%), 2 control (1.8%)) at baseline and 13 patients (8 mindfulness (4.8%), 5 control (5.5%)) at follow-up, fatigue, dizziness, shortness of breath, or pain due to pre-existing conditions were described while performing the 6MWT.

### Outcome analysis

**Fig 2:**
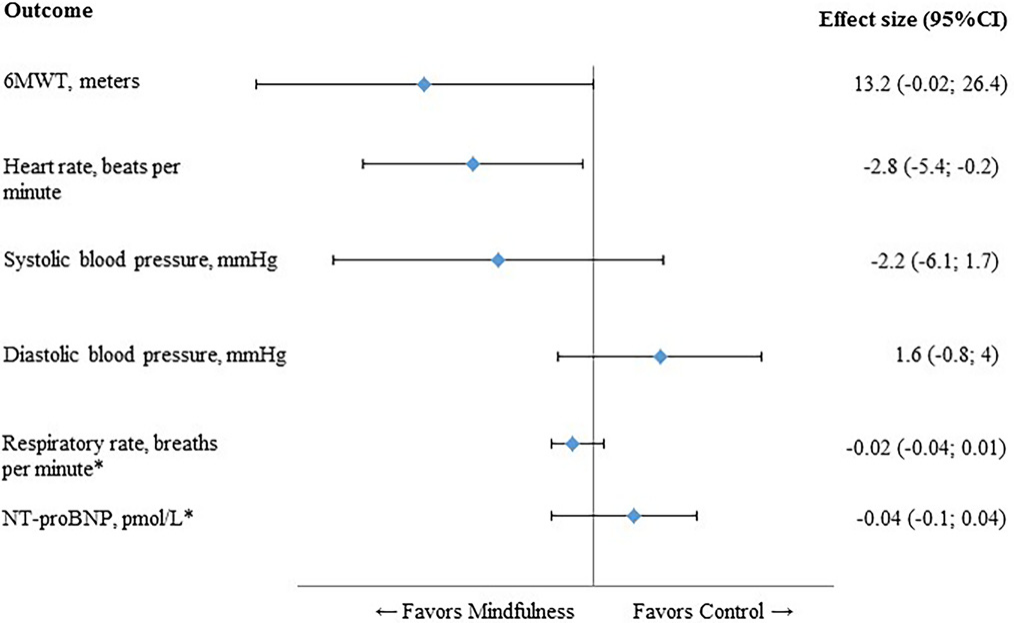
Forest plot of physiological outcomes. All values on the left of the Y-axis indicate a difference in favour of the mindfulness group. 6MWT, six-minute walk test; IC, confidence interval, NT-proBNP, N-terminal pro-brain natriuretic peptide; * Log-transformed scores.

At 12 weeks, the mindfulness group showed a notable improvement on their mean 6MWT, which was borderline significantly different compared with UC (p = 0.050) (Table 2). The intergroup comparison showed that heart rate was significantly lower in the mindfulness group (p = 0.033) (Table 2). Mean systolic and mean diastolic blood pressure decreased in the mindfulness and UC group, but no significant differences were found in the intergroup comparison (Table 2). The results of the physiological outcomes are summarized in Fig 2.

**Table 2:** Changes in outcomes at 12 weeks compared with baseline (intragroup effect) and differences between treatment groups (difference in delta’s, intergroup effect) on physiological and psychological outcomes. Intention-to-treat analyses.

Physiological outcomes	Treatment group	Delta 12-weeks vs baseline (intragroup)^a^ (mean, SD)	Effect Estimate (intergroup) ^b^	95% CI	p-value
**6MWT, meters**	Mindfulness	10.42 (49.0)	13.2	-0.02; 26.4	0.050
	UC	-4.0 (55.6)			
**Heart rate, beats/min**	Mindfulness	-2 (10.9)	-2.8	-5.4; -0.2	0.033
	UC	0.5 (9.0)			
**SBP, mmHg**	Mindfulness	-4.2 (15.4)	-2.2	-6.1; 1.7	0.268
	UC	-1.9 (15.5)			
**DBP, mmHg**	Mindfulness	-1.9 (8.9)	1.6	-0.8; 4.0	0.186
	UC	-3.4 (10.1)			
**Respiratory rate, breaths/minute** *	Mindfulness	-0.5 (3.6)	-0.02	-0.04; 0.01	0.189
	UC	-0.1 (4.0)			
**NT-proBNP, pmol/L** *	Mindfulness	0.3 (9.7)	-0.04	-0.1; 0.04	0.333
	UC	0.0 (11.10)			
**Psychological outcomes**					
**SF-36 PCS**	Mindfulness	0.5 (6.3)	-0.4	-2.0; 1.3	0.668
	UC	0.7 (6.7)			
**SF-36 MCS**	Mindfulness	0.2 (7.4)	0.74	-1.4; 2.8	0.489
	UC	1.2 (8.8)			
**VAS**	Mindfulness	0.4 (10.4)	-0.4	-3.0; 2.1	0.745
	UC	0.7 (9.3)			
**HADS Anxiety**	Mindfulness	-0.5 (3.2)	0.6	-0.2; 1.4	0.145
	UC	-0.9 (3.0)			
**HADS Depression**	Mindfulness	-0.5 (2.9)	-0.4	-1.1; 0.2	0.203
	UC	0.0 (2.3)			
**PSS**	Mindfulness	-2.4 (6.3)	-1.0	-2.7; 0.6	0.226
	UC	-0.9 (6.8)			
**PSSS12**	Mindfulness	0.6 (7.4)	0.4	-1.6: 2.4	0.685
	UC	0.1 (8.0)			

SD, standard deviation; SE, standard error; 6MWT, six-minute walk test; UC, usual care; SBP, systolic blood pressure; DBP, diastolic blood pressure; NT-proBNP, N-terminal pro-brain natriuretic peptide; SF-36, Short Form Health survey; PCS, physical component summary measure; MCS, mental component summary measure; VAS, visual analogue scale; HADS, hospital anxiety and depression scale; PSS, perceived stress score; PSSS12, perceived social support.

* Effect estimates are calculated from log-transformed scores.

^a^ Delta value (follow-up measurement minus baseline, intragroup effect) was calculated for those who attended the 12-week follow-up.

^b^ Linear mixed model analyses for repeated measurements for differences between treatment groups (intergroup effect) on the dependent variables (time X intervention effect).

Analyses showed no significant differences between the groups on the PCS and MCS of the SF-36 (Table 2). At 12 weeks, anxiety levels were lower than baseline scores in both the mindfulness and the UC group, but no significant differences were found in the intergroup comparison (Table 2).

**Fig 3:**
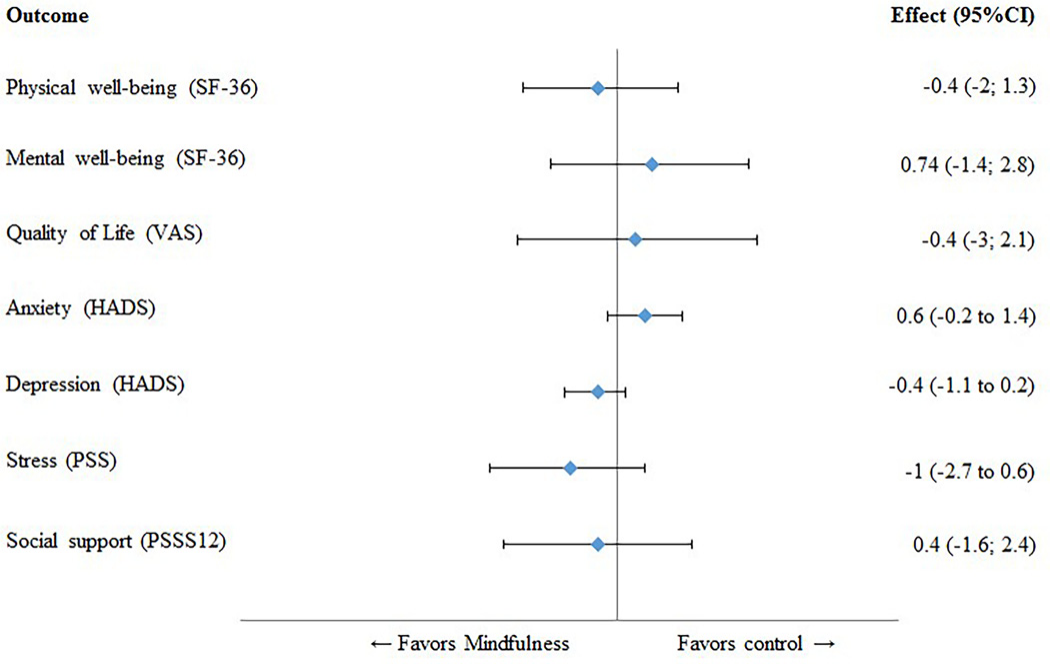
Forest plot of psychological outcomes. All values on the left of the Y-axis indicate a difference in favour of the mindfulness group. CI, confidence interval; SF-36, Short Form Health survey; VAS, visual analogue scale; HADS, hospital anxiety and depression scale; PSS, perceived stress score; PSSS12, perceived social support.

Depressive symptoms decreased at 12 weeks, but did not significantly differ between the groups. Neither perceived stress scores nor perceived social support were statistically significant different in the intergroup comparison (Table 2). No significant differences were found on adverse events. The results of the psychological outcomes are summarized in Fig 3.

The results of AT-analyses were comparable with the ITT analyses (Table 3).

**Table 3:** Changes in outcomes at 12 weeks compared with baseline (intragroup effect) and differences between treatment groups (intergroup effect) on physiological and psychological outcomes, as-treated analyses.

Physiological outcomes	Treatment group	Delta 12-weeks* (mean, SD)	Estimate†	95% CI	p
**6MWT, meters**	Mindfulness	9.36 (35.9)	10.6	-1.7; 23.0	0.091
	UC	-1.92 (51.7)			
**Heart rate, beats/min**	Mindfulness	-3.07 (11.7)	-3.4	-6.3; 0.4	0.027
	UC	0.47 (9.2)			
**SBP, mmHg**	Mindfulness	-5.17 (14.5)	-3.8	-8.0; 0.3	0.072
	UC	-1.50 (15.5)			
**DBP, mmHg**	Mindfulness	-2.34 (8.9)	0.8	-1.8; 3.5	0.524
	UC	-3.39 (10.1)			
**Respiratory rate, breaths/minute**	Mindfulness	-0.67 (3.5)	-0.7	-1.8; 0.3	0.170
	UC	-0.11 (4.1)			
**NT-proBNP, pmol/L** ‡	Mindfulness	1.03 (28.7)	-0.04	-0.2; 0.09	0.540
	UC	4.73 (21.7)			
**Psychological outcomes**					
**SF-36 PCS**	Mindfulness	0.7 (6.4)	0.13	-1.7; 2.0	0.893
	UC	0.4 (6.8)			
**SF-36 MCS**	Mindfulness	-0.05 (7.5)	1.2	-1.1; 3.5	0.302
	UC	1.5 (8.9)			
**VAS**	Mindfulness	0.4 (10.4)	-0.2	-3.0; 2.6	0.878
	UC	0.7 (9.3)			
**HADS Anxiety**	Mindfulness	-0.5 (3.2)	0.5	-0.4; 1.4	0.267
	UC	-0.9 (3.0)			
**HADS Depression**	Mindfulness	-0.5 (2.9)	-0.4	-1.2; 0.3	0.267
	UC	0.0 (2.3)			
**PSS**	Mindfulness	-2.4 (6.3)	-1.1	-3.0; 0.8	0.244
	UC	-0.9 (6.8)			
**PSSS12**	Mindfulness	0.6 (7.4)	0.5	-1.7; 2.6	0.670
	UC	0.1 (8.0)			

SD, standard deviation; SE, standard error; 6MWT, six-minute walk test; UC, usual care; SBP, systolic blood pressure; DBP, diastolic blood pressure; IQR, interquartile range; SE, standard error, NT.

proBNP, N-terminal pro-brain natriuretic peptide; SF-36, Short Form Health survey; VAS, visual analogue scale; HADS, hospital anxiety and depression scale; PSS, perceived stress score; PSSS12, perceived social support.

* Delta value (follow-up measurement minus baseline, intragroup effect) was calculated for those who attended the 12-week follow-up.

† Linear mixed model analyses for repeated measurements for differences between treatment groups on the dependent variables (time X intervention effect).

‡ Effect estimates are calculated from log-transformed scores.

### Cohen’s D

**Fig 4:**
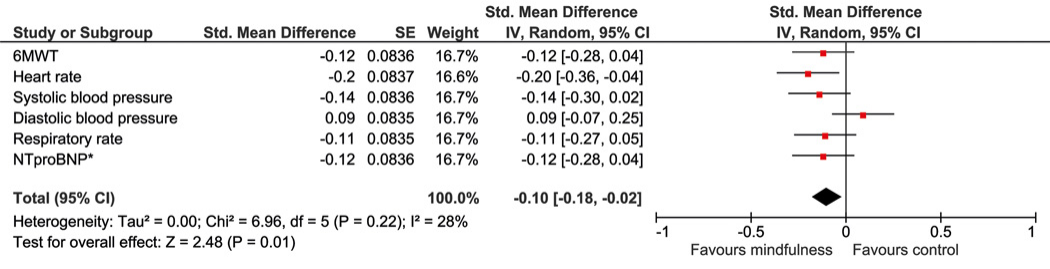
Forest plot showing the Intention-to-Treat Cohen’s D results of the effectiveness of the mindfulness intervention compared with usual care on the physiological outcomes. The width of the line indicates the 95%CI. All values lower than 0 indicate a significant difference in favour of the mindfulness group.

**Fig 5:**
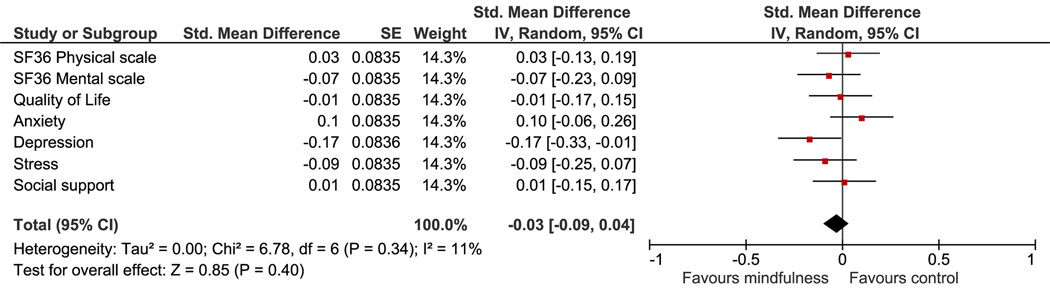
Forest plot showing the Intention-to-Treat Cohen’s D results of the effectiveness of the mindfulness intervention compared with usual care on the psychological outcomes. The width of the line indicates the 95%CI. All values lower than 0 indicate a significant difference in favour of the mindfulness group.

**Fig 6:**
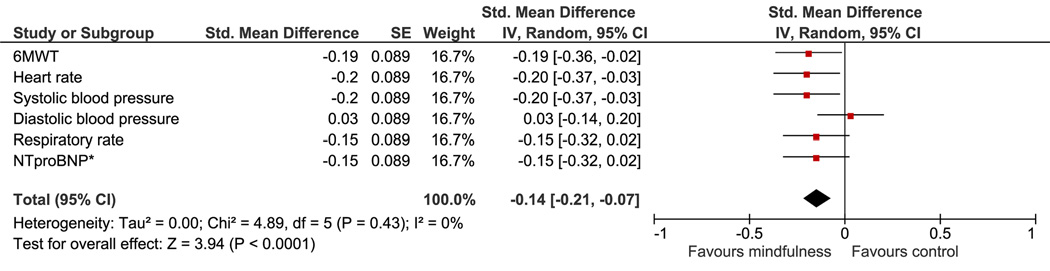
Forest plot showing the As-Treated Cohen’s D results of the effectiveness of the mindfulness intervention compared with usual care on the physiological outcomes. The width of the line indicates the 95%CI. All values lower than 0 indicate a significant difference in favour of the mindfulness group.

**Fig 7:**
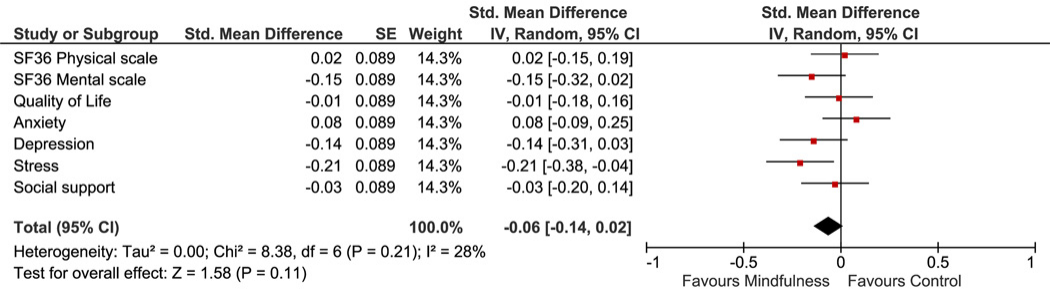
Forest plot showing the As-Treated Cohen’s D results of the effectiveness of the mindfulness intervention compared with usual care on the psychological outcomes. The width of the line indicates the 95%CI. All values lower than 0 indicate a significant difference in favour of the mindfulness group.

In order to compare different outcome measures, Cohen’s D effect sizes were calculated. In the intention-to-treat analyses (Figs 4 and 5), heart rate and depression showed, small, but significant improvement (D = 0.20, 95%CI 0.04 to 0.36 and d = 0.17, 95%CI 0.01 to 0.33 respectively). In the As-Treated analysis, exercise capacity, heart rate, systolic blood pressure and stress improved significantly, with small effect sizes ranging from D = 0.19 to D = 0.21 (Figs 6 and 7).

## Discussion

To our knowledge, this is the first randomized trial to evaluate the effectiveness of mindfulness training in patients with heart disease. By taking physiological parameters as its main outcome parameter, it is also an innovative study. On the primary endpoint–exercise capacity–we found a borderline significant but clinically small effect in favor of mindfulness. Heart rate also decreased significantly more with mindfulness training. Remarkably, no significant improvements were found on subjective outcome measures, although anxiety and depressive symptoms did decrease.

Limited exercise capacity is an important predictor for outcome for cardiac disease, and several studies have reported an association with survival.[17, 31–33] Since a decrease in physical performance is also an important predictor of adverse outcomes in patients with congenital heart disease, improving physical performance may be an important target of treatment. In recent years, cardiac rehabilitation programs, many of them conducted in patients with post-myocardial infarction, have had good results on total and cardiovascular mortality.[34, 35] Our results indicate that mindfulness training could be part of future treatment modalities intended to improve physical performance in heart disease patients. It remains to be shown whether this will also affect long-term outcome. Several epidemiological studies in patients with hypertension, acute coronary syndromes,[36] stable coronary heart disease[37] and heart failure[38] have shown that resting heart rate is a risk factor for cardiovascular and all-cause mortality. These epidemiological results suggest that the beneficial effect of mindfulness on heart rate demonstrated in our study is clinically meaningful.

To date, very few studies have evaluated mindfulness training in patients with cardiac disease. A pilot study that offered a brief mindfulness-based stress-reduction program to patients with, or at risk of, coronary artery disease[16] showed significant but moderate reductions on two psychological outcomes, depression (Cohen’s *d* = 0.54) and perceived stress (*d =* 0.68). Unlike in our study, the participants were not randomized and the intervention was fairly short (4-weeks). Two reports of the same study population showed that mindfulness-based stress-reduction mainly improved anxiety, emotional control and coping, rather than resting-stress hormones or physical functioning.[39, 40] Recently, a brief group-MBSR intervention in patients undergoing a percutaneous coronary intervention showed favorable effects on quality of life.[41] Additionally, anxiety, depression, and stress appeared to be influenced positively but only in the younger age group (<60 years).[41] Lastly, an individual MBSR training in patients with coronary heart disease showed significant reductions in anxiety, depression, perceived stress and the physiological parameters BP and BMI.[42] However, this study had limited power and was only performed in males. In contrast to previous reports, in our study psychological outcomes did not significantly improve by mindfulness training. Whereas in previous studies patients were often selected on the basis of reduced psychological well-being, in our study they were not. In fact, baseline psychological scores were similar to scores in the general population[43–47] implying that improvement was hardly possible (a ceiling effect). Also, our training was online without any personal contact, which probably resulted in smaller effects. There is increased interest in the effect of acceptance and commitment therapy, which has similarities with mindfulness training, focusing on the relationship between persons’ own thoughts and feelings that could potentially have a positive effect on several modifiable CVD risk factors.[48]

Similar to the ceiling effect for psychological outcomes, we observed a floor effect for blood pressure, as our patients had regular blood pressure monitoring and (extra) medication was given when necessary. Previous studies, some of which showed potential benefits on blood pressure, investigated populations whose blood pressures at baseline were higher than average.[7, 49]

Accumulating evidence suggests that mind and body do indeed show an interaction and that physiological changes are underlain by several neuro-humoral mechanisms. For example, in an extensive study of a framework in mind-body medicine, Benson and colleagues focused on the relaxation response as a core component in autonomic function and physical changes.[50, 51] It has been shown that, through emotions and thoughts, the autonomic nervous system is key in the brain-heart connection.[52] By working through the autonomic nervous system, mind-body practices can also benefit endothelial, neuroendocrine and immune function.[53–55] However, the mechanism between the mind and body is not merely unidirectional: several levels of the neuro-axis have been found to contribute to the “top-down and bottom-up mechanisms” in mind-body practices.[56]

To date, web-based mindfulness training studies have been limited to small studies on stress reduction. A study by Gluck et al.[57] reported a trend towards lower levels of stress. Two other studies on online mindfulness training showed not only that it was feasible to conduct online mindfulness training, but also that it was effective in reducing stress.[58, 59] It is important to emphasize that their study populations consisted of mainly females recruited from the healthy general population, and in one, no randomization was performed.

Limitations of the current study must be addressed. Our own study used neither a placebo nor a waiting list for the control group. We considered the placebo effect of the online training to be inherent to the active intervention: we evaluated the training and the control as they would be implemented in real-world practice and measured effectiveness rather than efficacy. We acknowledge that by doing so our study is pragmatic rather than explanatory. Our inclusion of the placebo effect as part of the mindfulness intervention compared with UC without placebo in the control group is further justified by the fact that no competing therapy exists. Every placebo online training similar to the intervention we could think of would likely have an unwanted beneficial effect in the control group.

Another limitation is that only 80.6% (n = 261) of the patients returned for follow-up. Reasons for trial discontinuation were not reported due to recommendations from the local ethics committee. A possible explanation for the follow-up rate is the fact that the intervention was not offered in a group-based setting. The impact of online training may be lower than that of personal or group training. While this would mean that the results of mindfulness therapy may therefore be even stronger in other settings, the easy accessibility of online training may have allowed better generalizability of the results, as patients could do the training in their own environment and fit it into a busy schedule. Although we monitored participants’ training activity, detailed adherence was difficult to assess and control for. Furthermore, ethical considerations prevented us from blinding patients to the nature of the intervention during the informed consent procedure and patients’ expectation of the interventions was not addressed. The control group was therefore aware that the online mindfulness training was available and that they were not receiving it. This could have led to selective follow-up but we found no significant difference between the groups at follow-up with regard to demographic and clinical variables. A placebo lifestyle intervention in the control group could underestimate the effectiveness of the active intervention compared to what can be expected in real-world practice. Thus, rather than comparing to a unrealistic placebo intervention, we considered the placebo effect as part-and-parcel of the procedure. In addition, we did not assess maintenance of blinding, but the inability to blind patients could have potentially led to unblinding of the investigators (outcome assessors), even though extensive precautions were made to limit this bias.

## Conclusions

Online mindfulness training is feasible in patients with heart disease and shows a small positive effect on exercise capacity and heart rate. The current study found no significant effect on psychological outcomes.

## Supporting Information

S1 Text Consort 2010 checklist of web-based mindfulness training in heart disease. (DOC)

S2 Text Trial Protocol. (DOC)

S1 Table Content web-based mindfulness training. (DOCX)
