# Supplementary material for: Web-Based Mindfulness Intervention in Heart Disease: A Randomized Controlled Trial
Source: PLoS One. 2015 Dec 7;10(12):e0143843. doi: 10.1371/journal.pone.0143843 (PMC4671576; doi:10.1371/journal.pone.0143843)
Supplement: S2 Text — (DOC) [file pone.0143843.s002.doc]

**Randomized controlled trial of mindfulness training as complementary therapy in adults with structural heart disease**

**RESEARCH PROTOCOL**

**February 23, 2012**

**PROTOCOL TITLE Randomized controlled trial of mindfulness training as complementary therapy in adults with structural heart disease**

| **Protocol ID** | **102480** |
| --- | --- |
| **Short title** | **RCT of mindfulness for structural heart disease** |
| **Version** | **3.0** |
| **Date** | **Feb 23, 2012** |
| **Coordinating investigator/project leader** | ***M.G. Myriam Hunink***  ***Jolien W. Roos-Hesselink*** |
| **Principal investigator(s) (hoofdonderzoeker/uitvoerder)** | ***M.G. Myriam Hunink***  ***Jolien W. Roos-Hesselink*** |
|  |  |
| **Sponsor (in Dutch: verrichter/opdrachtgever)** | ***Raad van Bestuur, represented by  Prof. dr. H. Pols, dean*** |
|  |  |
| **Independent physician(s)** | ***Prof. Dr. A.P. Kappetein*** |
|  |  |
|  |  |
| **Laboratory sites** | ***Klinisch Chemisch Laboratorium Erasmus MC*** |
|  |  |
|  |  |
| **Pharmacy** | ***Not applicable*** |
|  |  |

**PROTOCOL SIGNATURE SHEET**

| **Name** | **Signature** | **Date** |
| --- | --- | --- |
| **Sponsor or legal representative:**  ***Raad van Bestuur, represented by  Prof. dr. H. Pols, dean***  **For non-commercial research,**  **Head of Department:**  ***Prof.dr. F. Zijlstra*** |  |  |
| **Coordinating Investigator/Project leader/Principal Investigator:**  ***Jolien W. Roos-Hesselink, Prof Cardiology***  ***M.G. Myriam Hunink, Prof Clinical Epidemiology and Radiology*** |  |  |
|  |  |  |

**TABLE OF CONTENTS**

1. INTRODUCTION AND RATIONALE [9](#__RefHeading___Toc304302806)

2. OBJECTIVES [10](#__RefHeading___Toc304302807)

3. STUDY DESIGN [10](#__RefHeading___Toc304302808)

4. STUDY POPULATION [10](#__RefHeading___Toc304302809)

5. TREATMENT OF SUBJECTS [11](#__RefHeading___Toc304302810)

5.1 Investigational product/treatment [11](#__RefHeading___Toc304302811)

5.2 Use of co-intervention (if applicable) [12](#__RefHeading___Toc304302812)

5.3 Escape medication (if applicable) [12](#__RefHeading___Toc304302813)

6. INVESTIGATIONAL MEDICINAL PRODUCT [12](#__RefHeading___Toc304302814)

7. METHODS [12](#__RefHeading___Toc304302815)

7.1 Study parameters/endpoints [12](#__RefHeading___Toc304302816)

7.1.1 Main study parameter/endpoint [12](#__RefHeading___Toc304302817)

7.1.2 Secondary study parameters/endpoints [13](#__RefHeading___Toc304302818)

7.1.3 Other study parameters (if applicable) [13](#__RefHeading___Toc304302819)

7.2 Randomisation, blinding and treatment allocation [13](#__RefHeading___Toc304302820)

7.2.1 Flowchart [15](#__RefHeading___Toc304302821)

7.3 Study procedures [16](#__RefHeading___Toc304302822)

7.4 Withdrawal of individual subjects [17](#__RefHeading___Toc304302823)

7.4.1 Specific criteria for withdrawal (if applicable) [17](#__RefHeading___Toc304302824)

7.5 Replacement of individual subjects after withdrawal [17](#__RefHeading___Toc304302825)

7.6 Follow-up of subjects withdrawn from treatment [17](#__RefHeading___Toc304302826)

7.7 Premature termination of the study [17](#__RefHeading___Toc304302827)

8. SAFETY REPORTING [18](#__RefHeading___Toc304302828)

8.1 Section 10 WMO event [18](#__RefHeading___Toc304302829)

8.2 Adverse and serious adverse events [18](#__RefHeading___Toc304302830)

8.2.1 Suspected unexpected serious adverse reactions (SUSAR) [19](#__RefHeading___Toc304302831)

8.2.2 Annual safety report [19](#__RefHeading___Toc304302832)

8.3 Follow-up of adverse events [19](#__RefHeading___Toc304302833)

8.4 Data Safety Monitoring Board (DSMB) [19](#__RefHeading___Toc304302834)

9. STATISTICAL ANALYSIS [20](#__RefHeading___Toc304302835)

9.1 Descriptive statistics [20](#__RefHeading___Toc304302836)

9.2 Intention-to-treat and as-treated analysis [20](#__RefHeading___Toc304302837)

9.3 Univariable analysis [20](#__RefHeading___Toc304302838)

9.4 Multivariable analysis [21](#__RefHeading___Toc304302839)

9.5 Interim analysis (if applicable) [21](#__RefHeading___Toc304302840)

9.6 Economic Evaluation [21](#__RefHeading___Toc304302841)

10. ETHICAL CONSIDERATIONS [23](#__RefHeading___Toc304302842)

10.1 Regulation statement [23](#__RefHeading___Toc304302843)

10.2 Recruitment and consent [23](#__RefHeading___Toc304302844)

10.3 Objection by minors or incapacitated subjects (if applicable) [24](#__RefHeading___Toc304302845)

10.4 Benefits and risks assessment, group relatedness [24](#__RefHeading___Toc304302846)

10.5 Compensation for injury [25](#__RefHeading___Toc304302847)

10.6 Incentives (if applicable) [25](#__RefHeading___Toc304302848)

11. ADMINISTRATIVE ASPECTS AND PUBLICATION [26](#__RefHeading___Toc304302849)

11.1 Handling and storage of data and documents [26](#__RefHeading___Toc304302850)

11.2 Amendments [26](#__RefHeading___Toc304302851)

11.3 Annual progress report [26](#__RefHeading___Toc304302852)

11.4 End of study report [26](#__RefHeading___Toc304302853)

11.5 Public disclosure and publication policy [27](#__RefHeading___Toc304302854)

**LIST OF ABBREVIATIONS AND RELEVANT DEFINITIONS**

| **ABR** | **ABR form, General Assessment and Registration form, is the application form that is required for submission to the accredited Ethics Committee (In Dutch, ABR = Algemene Beoordeling en Registratie)** |
| --- | --- |
| **AE** | **Adverse Event** |
| **AR** | **Adverse Reaction** |
| **CA** | **Competent Authority** |
| **CCMO** | **Central Committee on Research Involving Human Subjects; in Dutch: Centrale Commissie Mensgebonden Onderzoek** |
| **CV** | **Curriculum Vitae** |
| **DSMB** | **Data Safety Monitoring Board** |
| **EU** | **European Union** |
| **EudraCT** | **European drug regulatory affairs Clinical Trials** |
| **GCP** | **Good Clinical Practice** |
| **IB** | **Investigator’s Brochure** |
| **IC** | **Informed Consent** |
| **IMP** | **Investigational Medicinal Product** |
| **IMPD** | **Investigational Medicinal Product Dossier** |
| **METC** | **Medical research ethics committee (MREC); in Dutch: medisch ethische toetsing commissie (METC)** |
| **(S)AE** | **(Serious) Adverse Event** |
| **SPC** | **Summary of Product Characteristics (in Dutch: officiële productinfomatie IB1-tekst)** |
| **Sponsor** | **The sponsor is the party that commissions the organisation or performance of the research, for example a pharmaceutical**  **company, academic hospital, scientific organisation or investigator. A party that provides funding for a study but does not commission it is not regarded as the sponsor, but referred to as a subsidising party.** |
| **SUSAR** | **Suspected Unexpected Serious Adverse Reaction** |
| **Wbp** | **Personal Data Protection Act (in Dutch: Wet Bescherming Persoonsgevens)** |
| **WMO** | **Medical Research Involving Human Subjects Act (in Dutch: Wet Medisch-wetenschappelijk Onderzoek met Mensen** |

**SUMMARY**

**Rationale:** Evidence is accumulating that mind-body therapies can be used as effective and safe adjuncts to medical treatment for a number of common clinical conditions. Some studies suggest that mindfulness training (also known as mindfulness-based stress reduction or mindfulness meditation) may have a beneficial clinical effect in patients with cardiovascular disease or those at increased cardiovascular risk. In addition, psychological problems and symptoms of depression and anxiety appear to be amenable to mindfulness meditation. Adult patients with structural heart disease have a high incidence of both physical (cardiac) and psychological problems and may therefore benefit from mindfulness training.

**Objective**: Objective of this study is to determine whether mindfulness training has beneficial clinical and psychological effects when provided as adjunct to usual care, and whether offering the training is effective and cost-effective, in adult patients with structural heart disease (congenital heart disease or cardiomyopathy).

**Study design:** Pragmatic randomized controlled single-blind trial.

**Study population:** Adults 18 years and older known to have structural heart disease. Exclusion criteria: patients in whom an operation or percutaneous intervention is planned, patients unable or unwilling to give informed consent, those without internet access, those unable to read or write Dutch. To demonstrate an improvement of 5% in the active intervention group vs 1% in the control group in exercise tolerance requires 330 patients.

**Intervention**: Mindfulness-training complementary to usual care. The mindfulness training consists of online sessions with assignments and practice supplemented with intermittent supportive email and text messages. The control group will receive usual care which includes lifestyle advice.

**Main study parameters/endpoints:** Primary outcome will be differences between the active intervention and control groups in the mean improvement, compared to baseline, of age- and sex expected exercise tolerance (6 minute walk test). The intention-to-treat analysis will address the question whether offering the training is effective. The as-treated analysis will evaluate the treatment effect depending on the degree of adherence. Secondary outcomes will be heart rate, blood pressure, and resting respiratory rate (markers of cardiac fitness), NTproBNP (a biomarker for heart failure), cumulative cortisol in 2 cm of hair taken from the scalp (cumulative measure of stress), a composite endpoint (all-cause mortality, heart failure, arrythmia, myocardial infarction, cardiac surgery, percutaneous cardiac intervention, electrical cardioversion), quality-of-life, psychological well-being, preference values, health care costs, and non-health care costs.

**Nature and extent of the burden and risks associated with participation, benefit and group relatedness:**

All patients will be given usual care which includes lifestyle advice. Patients in the active intervention group will be offered an online training for the duration of 12 weeks with email messages and assignments 1-3 times per week, followed by reminder messages once every 2-4 weeks for another 40 weeks. At baseline, 3-months, and 1 year all patients will undergo a 6 minute walk test and blood tests, one scalp hair will be taken, and they will be asked to fill out quality-of-life, psychological well-being, medical resource use and cost questionnaires.

# INTRODUCTION AND RATIONALE

Evidence is accumulating that mindfulness training and related mind-body therapies can be used as effective and safe adjuncts to medical treatment for a number of common clinical conditions including depression, insomnia, anxiety, post-traumatic stress, irritable bowel syndrome, nausea, pain, diabetes, hypertension, and cardiovascular disease [1-5]. Several publications report physiological effects of mind-body practices by modifying the response to chronic stress, including beneficial changes in respiration rate, heart rate, oxygen consumption, blood pressure, inflammatory markers, and brain activity. The biological pathway is through the autonomic nervous system. Neuroendocrine, endothelial, and immune system function all appear to be beneficially effected [3, 6-7]. Functional MRI performed during meditation or prayer demonstrates specific areas of increased or decreased activity [8-10]. Long-term structural changes in the brain have also been demonstrated among meditators and religious practitioners [11-12]. There are even data suggesting that gene expression is effected [13].

Some studies suggest that mindfulness training (also known as mindfulness-based stress reduction or mindfulness meditation) may have a beneficial clinical effect in patients with cardiovascular disease or those at increased cardiovascular risk [4, 14-15]. In addition, psychological problems and symptoms of depression and anxiety appear to be amenable to mindfulness meditation [3, 16-17]. Adult patients with structural heart disease have a high incidence of both physical (cardiac) and psychological problems and may therefore benefit from mindfulness training [18-19].

# OBJECTIVES

Primary Objective of this study is to determine whether mindfulness training has beneficial clinical and psychological effects in adult patients with structural heart disease (congenital heart disease or cardiomyopathy).

Secondary Objective is to determine whether offering mindfulness training is effective and cost-effective in adult patients with structural heart disease.

# STUDY DESIGN

Pragmatic randomized controlled single-blind trial.

Duration: 12 months

Setting: outpatient clinic of an academic hospital

# STUDY POPULATION

**Population (base)**
The source population are adult patients attending the ErasmusMC cardiology outpatient clinic for follow-up and monitoring of their structural heart disease, including those with congenital heart disease and those with cardiomyopathy.

**Inclusion criteria**

1. Adults 18 to 65 years old known to have structural heart disease, including congenital heart disease and cardiomyopathy.

**Exclusion criteria**

1. planned operation or percutaneous intervention
2. inability or unwillingness to give informed consent
3. inability to understand Dutch, inability to read or write Dutch
4. no internet access or no email or no cell phone.
5. patients who do not fill out their baseline questionnaires or do not show up for the scheduled baseline tests

**Sample size calculation**

To demonstrate an improvement of 5% in the active intervention group vs 1% in the control group in exercise tolerance (expressed in percentage exercise tolerance compared to what can be expected given the patient’s age and sex) would require 99 patients in the control group and 198 in the active intervention group (SD10%, alpha=0.05, power=0.90, ratio experimental to controls=2). Even if only 50% of patients in the experimental group adhere to the training, we would in the as-treated analysis compare three groups of each 99 patients, which would give us a power of 0.80. To account for non-adherence and loss to follow-up a total of 330 patients will be randomized. This number of patients will be sufficient to demonstrate a smaller difference (5% vs 2%) in a repeated measurements analysis with a power of 75-90% (2 follow-up measurements, correlation between follow up measurements=0.70, correlation between baseline & follow-up=0.50).

# TREATMENT OF SUBJECTS

## Investigational product/treatment

Intervention:

The intervention is a mindfulness training and consists of a structured standardized online program, which has been provided to clients in the general population (http://www.psychologiemagazine.nl/web/Trainingen/Online-training-mindfulness.htm). Every 3 days the patient receives an email message with a link to a website where they are offered text explanations, video clips, sound tracks with mindfulness exercises, breathing exercises, assignments that need to be filled out, and practice suggestions for the coming few days. The online program is further supported by emails and intermittent text messages on the patient’s cell phone. The online training is currently 8 weeks. It will be extended to 12 weeks followed by reminder emails, sms-messages, and suggestions for continuing practice every 2-4 weeks thereafter until a year after the beginning. The low cost of the online programme (€ 30,-) gives the program the potential of being a cost effective intervention. Patients will not be charged for the online program. They will receive a personal code from Psychologie Magazine to start the online mindfulness training. Adherence to the intervention will be monitored by documenting whether the questions have been filled out.

In addition, all patients will receive usual care just like patients in the control group.

Control:

The control group will receive usual care which consists of regular outpatient visits, lifestyle advice regarding healthy nutrition, smoking cessation, physical activity, and stress reduction, and medical therapy and procedures if indicated.

Justification: See section 7.2

## Use of co-intervention (if applicable)

All patients will receive usual care including lifestyle advice for their structural heart disease.

## Escape medication (if applicable)

Not applicable

# INVESTIGATIONAL MEDICINAL PRODUCT

Not applicable

# METHODS

## Study parameters/endpoints

### Main study parameter/endpoint

It is most likely that the mindfulness training will result in an improved quality of life. In this study we set out to search for an even more ambitious goal, and that is to find effects on physical variables. The 6 minute walk test has been chosen as primary outcome since it is a measure of cardiac and overall physical fitness. We expect that the mindfulness training will improve cardiac and overall physical fitness through the mindful breathing exercises, by encouraging a healthy lifestyle, and by reducing chronic anxiety and stress levels.

Primary outcome will be differences between the active intervention and control groups in the mean improvement at 3 months and 1 year, compared to baseline, of age- and sex expected exercise tolerance.

Exercise tolerance will be measured at baseline, 3 months and 1 year with the 6-minute-walk test (in which the distance is measured that a patient can walk within 6 minutes).

### Secondary study parameters/endpoints

Key secondary outcome (measured at baseline, 3 months, 1 year):

- quality-of-life (SF36), using the mental- and physical composite outcome scales

Other secondary outcomes (measured at baseline, 3 months, 1 year):

- heart rate, blood pressure, resting respiratory rate (markers of cardiac fitness)
- NTproBNP (a biomarker for heart failure)
- cumulative cortisol in 2 cm of hair taken from the scalp (cumulative measure of stress)
- composite endpoint (all-cause mortality, heart failure, arrythmia, cardiac surgery, percutaneous cardiac intervention, electrical cardioversion)
- psychological well-being (Hospital Anxiety Depression questionnaire)
- preference values (societal EuroQol EQ-5D-5L values, societal SF-6D values obtained from the SF-36, VAS rating scale [Philip Moons version])
- Social support (perceived social support scale 12 Blumenthal PSSS12)
- Medical consumption of health-care use
- health care costs
- related non-health care costs (Tic-P)

### Other study parameters (if applicable)

We will document and adjust for baseline levels of traditional cardiovascular risk factors (blood pressure, smoking, lipids levels, body mass index), pre-existing anxiety and depression at baseline, and social economic status.
In the active intervention group we will monitor adherence to the online mindfulness training through the frequency of logging onto the website. In both active intervention and control group we will monitor participation in other mindfulness-based programs, practice of mindfulness-based exercises, and use of complementary care (through a modified Tic-P questionnaire).

## Randomisation, blinding and treatment allocation

(see flowchart on next page)

All potentially eligible patients are asked to participate in a randomized controlled trial. Written informed consent is obtained for extra data collection alongside usual care and for randomization to one of two healthy lifestyle programs. Patients are explained that mindfulness training is the active intervention and that they will have a 50% chance to be randomized to the active intervention group. The general principle of mindfulness is explained and patients are informed that the training is a web-based online training with exercises that they need to do themselves. Further details about the training and how to access the website and their login will be given after randomization.

Patients will be randomized to either offering online mindfulness training plus usual care (active intervention) vs usual care only (control) (which includes advice about a healthy lifestyle). Patients will be randomly assigned to the active intervention (offering mindfulness training plus usual care) vs control (usual care) in the ratio 2:1 to compensate for non-adherence with the intervention. Randomization will be performed by computer using dedicated software designed for this purpose. Block randomization will be used. The block size will be undisclosed until trial completion.

Patients will not be blinded to the intervention itself since they are actively participating in it. They will, however, be asked to avoid describing their training during follow-up visits to other patients, physicians, and investigators in order to ensure single-blinding. To blind investigators, the treatment allocation will remain undisclosed to the observer assessing outcomes and during data analysis. We will confirm observer blinding by asking the assessor of the exercise tolerance tests which treatment arm he/she thinks the patient has been allocated to.

### Flowchart

Justification of no placebo online training in the control group and single-blinded design:

(A1) We consider the placebo effect of the online training as being part of the active intervention as it would be implemented in real-world practice which implies that the study will measure effectiveness rather than efficacy and that it is pragmatic rather than explanatory.

(A2) The inclusion of the placebo effect as part of the active intervention compared to usual care without placebo in the control group is further justifiable as no competing therapy exists.

(A3) Any placebo online training that we develop is likely to have some beneficial effect, due to non-specific therapeutic mechanisms like structure, hope and working alliance. Such beneficial effect in the control group will reduce the measured difference in effectiveness and would not be an accurate reflection of what can be expected in real-world practice.

(A4) Blinding of patients to the intervention itself is impossible since they are themselves actively participating in the online training.

## Study procedures

At baseline, 3-months, and 1 year all patients will undergo exercise tolerance testing, measurement of heart rate, blood pressure and resting respiratory rate, cortisol measurement in 2 cm of scalp hair, and NTproBNP measurement and they will be asked to fill out questionnaires concerning quality-of-life, psychological well-being, medical resource use, and non-health care costs. Exercise tolerance will be measured with the 6-minute-walk test (in which the distance is measured that a patient can walk within 6 minutes).

NTproBNP will be determined in a blood sample. These samples will be coded in storage. Cumulative cortisol will be measured in 2 cm of one hair taken from the scalp. Clinical follow-up will provide data required for the composite endpoint.

Quality-of-life will be evaluated with the SF36. Psychological well-being will be determined with the Hospital Anxiety Depression Scale (HADS).

Preference values with be evaluated with the EuroQol EQ-5D-5L (societal values), the SF-6D from the SF-36 answers, and the VAS rating scale (patient values). Social support will be measured with the perceived social support scale 12 (PSSS12). Medical consumption will be measured with the Tic-P.Health care costs which will be determined on the basis of medical resource use multiplied by the actual or reimbursed costs per resource. Related non-health care costs will be determined using questionnaires (Tic-P) and will consist of the time costs for undergoing procedures, time costs for self-care including the mindfulness-training, the costs for support and care, costs for complementary care, and friction costs.

## Withdrawal of individual subjects

Subjects can leave the study at any time for any reason if they wish to do so without any consequences. The investigator can decide to withdraw a subject from the study for urgent medical reasons. If a patient decides to withdraw, the investigator will ask the patient consent for including the data collected thus far in the analysis.

A potential problem would arise if a large group of patients randomized to the active intervention are non-adherent to the active intervention. Nevertheless, non-adherence is considered part of what can be expected in the future when such an intervention is implemented in everyday practice and thus effectiveness rather than efficacy is measured.

### Specific criteria for withdrawal (if applicable)

None

## Replacement of individual subjects after withdrawal

None

## Follow-up of subjects withdrawn from treatment

None

## Premature termination of the study

Since we are investigating a complementary no-risk lifestyle intervention there will be no reason for premature termination of the study.

# SAFETY REPORTING

## Section 10 WMO event

In accordance to section 10, subsection 1, of the WMO, the investigator will inform the subjects and the reviewing accredited METC if anything occurs, on the basis of which it appears that the disadvantages of participation may be significantly greater than was foreseen in the research proposal. The study will be suspended pending further review by the accredited METC, except insofar as suspension would jeopardise the subjects’ health. The investigator will take care that all subjects are kept informed.

## Adverse and serious adverse events

Adverse events are defined as any undesirable experience occurring to a subject during the study, whether or not considered related to the experimental treatment]. All adverse events reported spontaneously by the subject or observed by the investiga­tor or his staff will be recorded.

A serious adverse event is any untoward medical occurrence or effect that at any dose:

- results in death;
- is life threatening (at the time of the event);
- requires hospitalisation or prolongation of existing inpatients’ hospitalisation;
- results in persistent or significant disability or incapacity;
- is a congenital anomaly or birth defect;
- is a new event of the trial likely to affect the safety of the subjects, such as an unexpected outcome of an adverse reaction, lack of efficacy of an IMP used for the treatment of a life threatening disease, major safety finding from a newly completed animal study, etc.

All SAEs will be reported through the web portal *ToetsingOnline* to the accredited METC that approved the protocol, within 15 days after the sponsor has first knowledge of the serious adverse reactions..

SAEs that result in death or are life threatening should be reported expedited. The expedited reporting will occur not later than 7 days after the responsible investigator has first knowledge of the adverse reaction. This is for a preliminary report with another 8 days for completion of the report.

### Suspected unexpected serious adverse reactions (SUSAR)

Not applicable because the intervention is not an investigational medicinal product

### Annual safety report

Not applicable because the intervention is not an investigational medicinal product

## Follow-up of adverse events

All adverse events will be followed until they have abated, or until a stable situation has been reached. Depending on the event, follow up may require additional tests or medical procedures as indicated, and/or referral to the general physician or a medical specialist.

## Data Safety Monitoring Board (DSMB)

The general DSMB of the Erasmus MC / LUMC will be asked to oversee this study. The advice(s) of the DSMB will be notified upon receipt by the sponsor to the METC that approved the protocol. With this notification a statement will be included indicating whether the advice will be followed.

# STATISTICAL ANALYSIS

## Descriptive statistics

Descriptive statistics will be calculated to describe the study population and to determine if any chance differences exist between the groups. Formal tests for differences will not be performed since we trust that the randomization procedure will be properly performed. Missing values will be imputed with a multiple imputation approach. The results will be tabulated.

## Intention-to-treat and as-treated analysis

An intention-to-treat analysis will be performed to address whether offering mindfulness training is effective and cost-effective. Patients that are non-adherent to the offered training (cross-overs to usual care) will be analyzed as part of the active intervention group. Similarly, control patients who seek interventions on their own will be analyzed in the usual care group to which they were allocated.

An as-treated analysis will be performed to address whether mindfulness training is beneficial if actually performed and the treatment effect depending on the degree of adherence. The usual care group will consist of the control group. In the active intervention group, adherence to the intervention is monitored (see ‘’Intervention’’) and completion of sufficient number of training modules will be the criterion to determine which patients in the active intervention arm are included in the ‘’on-treatment’’ group. The ‘’on-treatment’’ group will consist of those patients who complete 50% or more of the exercises in the first 12 weeks. Patients who start the mindfulness training but complete less than 50% of the assignments in the first 12 weeks will be considered non-adherent and analyzed separately as the “partially treated’’ group. We will also evaluate outcome as function of the proportion of completed exercises as a continuous variable adjusting for potential confouders in a multivariable model. Finally, we will use the method developed by Robins and Tsiatis that corrects for non-compliance in randomized trials using rank preserving structural failure time models [25-26].

## Univariable analysis

Short-term results (3 months) and mid-term results (1 year) will be calculated. Differences in mean improvements between the groups will be calculated for all outcome measures expressed on a continuous scale. Analysis with repeated measurement methods (mixed models) will be performed to account for multiple measurements performed during follow-up and for missing values.

Survival analysis will be performed with a failure being an event as defined according to the composite endpoint.

## Multivariable analysis

Multivariable analysis will be performed to adjust for any chance differences in risk factors at baseline that can clinically be expected to affect the outcome, for congenital heart disease vs cardiomyopathy, and for socio-economic status and to test for interaction effects. Furthermore, we will test for a differential effect on outcome depending on the level of pre-existing anxiety and depression.

## Interim analysis (if applicable)

No interim analysis will be performed.

## Economic Evaluation

If the 3 months and 1-year results indicate effectiveness of the mindfulness training, we will develop a Markov decision model to calculate long-term effectiveness, costs, and cost-effectiveness of offering mindfulness training to patients with structural heart disease. The comparison strategy will be usual care. We will perform the cost-effectiveness analysis from the health care and societal perspective considering a lifetime timeframe.

The model will consider different classes of heart failure, cardiovascular risk factors, cardiac events, quality of life, and costs. Event rates, risk factor changes, quality-of-life, health resource utilization, and cost data obtained from the clinical study will be integrated in the models. All co-investigators will be asked to review the model assumptions and inputs prior to performing the analysis in order to avoid bias. The economic evaluation will be performed in accordance with the Dutch recommendations for cost effectiveness analyses and we will explore UK and USA recommendations and costs in a sensitivity analysis.

Quality of life will be based on results from the trial where possible and where necessary from systematic reviews and Quality of Life catalogues available on the web. Costs that will be considered include direct medical costs (health-care costs for mind-body program, events, admissions, rehabilitation after an event, nursing home care for long-term disability), patient costs (out-of-pocket expenses for the mind-body program, time and travel costs associated with the program), and productivity losses (friction costs). Health-care costs will be estimated by multiplying health-care resource utilization with the cost per unit of resource. Future costs and life years will be discounted.

With the decision model we will calculate the effectiveness, the costs, the incremental cost-effectiveness ratios, the net health benefit, and the net monetary benefit for a number of willingness-to-pay thresholds. We will perform extensive (probabilistic) sensitivity analysis, threshold analysis, and value of information analysis.

# ETHICAL CONSIDERATIONS

## Regulation statement

The study will be conducted according to the principles of the Declaration of Helsinki (www.wma.net) and in accordance with the Medical Research Involving Human Subjects Act (WMO).

## Recruitment and consent

*The patient information letter and informed consent form should be attached as a separate document.*

1. Potentially eligible patients will be sought by considering every patient that is scheduled for a routine outpatient visit for periodic follow-up of congenital heart disease or cardiomyopathy.
2. Four weeks prior to the patient’s routine outpatient visit, the patient will be informed by post with written information about the study. (See attached patient information letter)
3. Two weeks later the written information will be followed by a telephone call by a junior doctor (researcher / PhD student) in which oral information will be given and any questions that the patient may have about the study will be addressed. Eligibility criteria will be tested at that time. In addition, intention-to-participate will be verified. If the patient is eligible and intends to participate in the study, exercise testing and lab work will be scheduled to take place as part of the patient’s next outpatient visit 2 weeks later.
4. During the outpatient visit, the junior doctor will address any remaining questions that the patient may have about the study and obtain written informed consent. (see attached informed consent form). If the patient refuses participation at that time the baseline measurements will not be performed and the scheduled tests will be cancelled.
5. If the patient consents to be included, the junior doctor will perform the 6 minute walk test, measure heart rate, blood pressure and resting respiratory rate, take one scalp hair, guide the patient through the questionnaires (to be filled out on a computer during the outpatient visit), and guide them to the laboratory for blood work.
6. The outpatient visit will be performed as usual. The treating doctor will give lifestyle advice as usual.
7. Following the outpatient visit and baseline measurements the patient will be randomized by computer and, if randomized to the active intervention, he/she will receive an email and letter with the appropriate link to the online training. The clinical researchers on the study will not be involved in this step and will therefore be unaware of the treatment assignment.

## Objection by minors or incapacitated subjects (if applicable)

Not applicable

## Benefits and risks assessment, group relatedness

All patients will be given usual care which includes lifestyle advice. Patients in the active intervention group will, in addition, be offered an online training for the duration of 12 weeks with email messages and assignments 1-3 times per week, followed by reminder messages once every 2-4 weeks for another 40 weeks. At baseline, 3-months, and 1 year all patients will undergo 6 minute walk test and blood tests, one scalp hair will be taken, and they will be asked to fill out quality-of-life, psychological well-being, medical resource use and cost questionnaires.

There is no risk of the active intervention. We anticipate that the active intervention will improve patients’ psychological well-being and physical fitness.

## Compensation for injury

The sponsor/investigator has a liability insurance which is in accordance with article 7, subsection 6 of the WMO.

The sponsor (also) has an insurance which is in accordance with the legal requirements in the Netherlands (Article 7 WMO and the Measure regarding Compulsory Insurance for Clinical Research in Humans of 23th June 2003). This insurance provides cover for damage to research subjects through injury or death caused by the study.

1. € 450.000,-- (i.e. four hundred and fifty thousand Euro) for death or injury for each subject who participates in the Research;
2. € 3.500.000,-- (i.e. three million five hundred thousand Euro) for death or injury for all subjects who participate in the Research;
3. € 5.000.000,-- (i.e. five million Euro) for the total damage incurred by the organisation for all damage disclosed by scientific research for the Sponsor as ‘verrichter’ in the meaning of said Act in each year of insurance coverage.

The insurance applies to the damage that becomes apparent during the study or within 4 years after the end of the study.

## Incentives (if applicable)

Patients who participate in the active intervention will be reimbursed for the costs of the online training (30 euro).

# ADMINISTRATIVE ASPECTS AND PUBLICATION

## Handling and storage of data and documents

Data will be handled confidentially and during the analysis phase anonymously. During the data collection phase it is necessary to be able to trace data to an individual subject so as to be able to complete the data collection. Subjects will get a study identification code and the list of codes will be used to link the data to the subject. The code will not be based on the patient initials and birth-date. The key to the code will be safeguarded by the investigator. The handling of personal data will comply with the Dutch Personal Data Protection Act (in Dutch: De Wet Bescherming Persoonsgegevens, Wbp).

## Amendments

All substantial amendments will be notified to the accredited METC. A ‘substantial amendment’ is defined as an amendment to the terms of the METC application, or to the protocol or any other supporting documentation, that is likely to affect to a significant degree:

- the safety or physical or mental integrity of the subjects of the trial;
- the scientific value of the trial;
- the conduct or management of the trial; or
- the quality or safety of any intervention used in the trial.

Non-substantial amendments (typing errors and administrative changes like changes in names, telephone numbers and other contact details of involved persons mentioned in the submitted study documentation) will not be notified to the accredited METC, but will be recorded and filed by the sponsor.

## Annual progress report

The sponsor/investigator will submit a summary of the progress of the trial to the accredited METC once a year. Information will be provided on the date of inclusion of the first subject, numbers of subjects included and numbers of subjects that have completed the trial, serious adverse events/ serious adverse reactions, other problems, and amendments.

## End of study report

The investigator will notify the accredited METC of the end of the study within a period of 8 weeks. The end of the study is defined as the last patient’s last visit.

In case the study is ended prematurely, the investigator will notify the accredited METC, including the reasons for the premature termination.

 Within one year after the end of the study, the investigator/sponsor will submit a final study report with the results of the study, including any publications/abstracts of the study, to the accredited METC.

## Public disclosure and publication policy

The results of the study will be disclosed unreservedly. In accordance with the requirements of the International Committee of Medical Journal Editors, as a condition for consideration for publication in a peer-reviewed journal, the trial will be registered in a public trial registry [ <http://clinicaltrials.gov/> and http://[www.clinicaltrialsregister.eu/](http://www.clinicaltrialsregister.eu/) ].

The results will be analyzed and reported in scientific publications to be submitted to peer-reviewed journals. In addition, the papers will be included in a PhD thesis. All authors on the papers must fulfill the criteria for authorship. Prof. Roos-Hesselink and Prof. Hunink will share responsibility for the study and for supervising the PhD student and all credits for the study will be shared equally between their research groups (Congenital Heart Disease and Clinical Epidemiology resp.)

REFERENCES

1. Ludwig, D.S. and J. Kabat-Zinn, *Mindfulness in medicine.* Jama, 2008. **300**(11): p. 1350-2.

2. Ospina, M.B., et al., *Meditation practices for health: state of the research.* Evid Rep Technol Assess (Full Rep), 2007(155): p. 1-263.

3. Chiesa, A. and A. Serretti, *A systematic review of neurobiological and clinical features of mindfulness meditations.* Psychol Med, 2009: p. 1-14.

4. Chiesa, A. and A. Serretti, *Mindfulness-based stress reduction for stress management in healthy people: a review and meta-analysis.* J Altern Complement Med, 2009. **15**(5): p. 593-600.

5. Innes, K.E., T.K. Selfe, and A. Vishnu, *Mind-body therapies for menopausal symptoms: a systematic review.* Maturitas, 2010. **66**(2): p. 135-49.

6. Benson, H., *The relaxation response: therapeutic effect.* Science, 1997. **278**(5344): p. 1694-5.

7. Dod, H.S., et al., *Effect of intensive lifestyle changes on endothelial function and on inflammatory markers of atherosclerosis.* Am J Cardiol, 2010. **105**(3): p. 362-7.

8. Lazar, S.W., et al., *Functional brain mapping of the relaxation response and meditation.* Neuroreport, 2000. **11**(7): p. 1581-5.

9. Shimomura, T., et al., *Functional brain mapping during recitation of Buddhist scriptures and repetition of the Namu Amida Butsu: a study in experienced Japanese monks.* Turk Neurosurg, 2008. **18**(2): p. 134-41.

10. Harris, S., et al., *The neural correlates of religious and nonreligious belief.* PLoS One, 2009. **4**(10): p. e0007272.

11. Lazar, S.W., et al., *Meditation experience is associated with increased cortical thickness.* Neuroreport, 2005. **16**(17): p. 1893-7.

12. Kapogiannis, D., et al., *Neuroanatomical variability of religiosity.* PLoS One, 2009. **4**(9): p. e7180.

13. Dusek, J.A., et al., *Genomic counter-stress changes induced by the relaxation response.* PLoS One, 2008. **3**(7): p. e2576.

14. Paul-Labrador, M., et al., *Effects of a randomized controlled trial of transcendental meditation on components of the metabolic syndrome in subjects with coronary heart disease.* Arch Intern Med, 2006. **166**(11): p. 1218-24.

15. Arthur, H.M., C. Patterson, and J.A. Stone, *The role of complementary and alternative therapies in cardiac rehabilitation: a systematic evaluation.* Eur J Cardiovasc Prev Rehabil, 2006. **13**(1): p. 3-9.

16. Sullivan, M.J., et al., *The Support, Education, and Research in Chronic Heart Failure Study (SEARCH): a mindfulness-based psychoeducational intervention improves depression and clinical symptoms in patients with chronic heart failure.* Am Heart J, 2009. **157**(1): p. 84-90.

17. Hofmann SG, S.A., Witt AA, Oh D, *The Effect of Mindfulness-Based Therapy on Anxiety and Depression: A Meta-Analytic Review.* J Consult Clin Psychol, 2010. **78(2)**: p. 169–183.

18. Pignay-Demaria, V., et al., *Depression and anxiety and outcomes of coronary artery bypass surgery.* Ann Thorac Surg, 2003. **75**(1): p. 314-21.

19. Swenson, J.R. and J.J. Clinch, *Assessment of quality of life in patients with cardiac disease: the role of psychosomatic medicine.* J Psychosom Res, 2000. **48**(4-5): p. 405-15.

20. Campbell, R., et al., *Adapting the randomized consent (Zelen) design for trials of behavioural interventions for chronic disease: feasibility study.* J Health Serv Res Policy, 2005. **10**(4): p. 220-5.

21. Homer, C.S., *Using the Zelen design in randomized controlled trials: debates and controversies.* J Adv Nurs, 2002. **38**(2): p. 200-7.

22. Huibers, M.J., et al., *An alternative trial design to overcome validity and recruitment problems in primary care research.* Fam Pract, 2004. **21**(2): p. 213-8.

23. Zelen, M., *Randomized consent designs for clinical trials: an update.* Stat Med, 1990. **9**(6): p. 645-56.

24. Piantadosi, S., *Clinical Trials: a methodologic perspective*. Second ed. 2005: Wiley & Sons, Inc.

25. Robins, J.M. and T. A.A., *Correcting for non-compliance in randomized trials using rank preserving structural failure time models.* Communications in Statistics - Theory and Methods, 1991. **20**(8): p. 2609-2631.

26. White, I.R., S. Walker, and A. Babiker, *strbee: Randomization-based efficacy estimator.* The Stata Journal, 2002. **2**(2): p. 140-150.
