# Supplementary material for: Web-Based Mindfulness Intervention in Heart Disease: A Randomized Controlled Trial
Source: PLoS One. 2015 Dec 7;10(12):e0143843. doi: 10.1371/journal.pone.0143843 (PMC4671576; doi:10.1371/journal.pone.0143843)
Supplement: S1 Table — (DOCX) [file pone.0143843.s003.docx]

**S3 Table.** Content web-based mindfulness training

Content of online training Mindfulness for a Healthier Heart.

| **Week** | **Content of the training** |
| --- | --- |
| **1** | **Do you really feel alive, don’t watch but see** |
|  | - Introduction film by Edel Maex - Likert-scale ‘really living’ - Exercise focused attention, watch your computer mindfully - 15 minutes Bodyscan - Observing pictures - What deserves more attention in your life? |
| **Homework** | - Weekly 15 minutes Bodyscan |
| **2** | **Hear what people say, avoid the automatic pilot** |
|  | - Adjust questioning and listening behavior - Short film on living separated from your surroundings - List daily activities performed on automatic pilot |
| **Homework** | - Execute 3 automatic activities with mindfulness |
| **3** | **Learn to meditate** |
|  | - Short film Edel Maex - 15 min sitting meditation - Planning sitting meditation - Remember what deserves more attention? |
| **Homework** | - Daily 15 minutes sitting meditation |
| **4** | **Dealing with stress, today is an important day** |
|  | - Short film stress reaction - 3-minute breathing space - Description of current day |
| **5** | **Learn to stop aware, your own mindfulness-reminder** |
|  | - Inventory stopping with current activity - Short film with quotes - Short film about death |
| **Homework** | - 3x per day 20 minutes stopping current activity; with awareness |
| **6** | **You are not your thoughts** |
|  | - Thoughts are not facts, you are not your thoughts - Difficult memories - Being compassionate |
| **7** | **The art of living consciously** |
|  | - Intention to give more love to… - Attention to seasons/ environment |
| **8** | **Mindfulness for the rest of your life** |
|  | - Likert-scale feeling of being really alive - Evaluation of what you have learned - Short film of laughing people |
| **9** | **Staying Mindful** |
|  | - 6-minute meditation practice - Planning mindfulness |
| **10** | **Rewrite your personal book of laws** |
|  | - What rules do you impose on yourself? - Which rules do you really have to adhere to? - What do you allow yourself to do? |
| **11** | **Mindful eating** |
|  | - Short film about eating in between daily chores - Assignment eating breakfast, lunch and dinner mindfully - Note your experiences |
| **12** | **Yoga for your heart** |
|  | - Yoga 30 minutes (instruction film) - Likert-scale experiences - Planning Yoga into your schedule |
